# Chemerin Is the Adipokine Linked with Endothelin-Dependent Vasoconstriction in Human Obesity

**DOI:** 10.3390/biomedicines13092131

**Published:** 2025-08-31

**Authors:** Francesca Schinzari, Rossella Montenero, Carmine Cardillo, Manfredi Tesauro

**Affiliations:** 1 Department of Aging, Policlinico A. Gemelli IRCCS, 00168 Rome, Italy; 2 Department of Systems Medicine, Tor Vergata University, 00133 Rome, Italy; 3 Department of Translational Medicine and Surgery, Università Cattolica del Sacro Cuore, Largo F. Vito 1, 00168 Rome, Italy

**Keywords:** chemerin, adipokines, obesity, endothelin, vasoconstriction

## Abstract

**Background/Objectives**: The remodeling of adipose tissue occurring in obesity is associated with dysregulated production of various adipokines with vasoactive properties. Among the local mediators physiologically involved in vascular homeostasis, the endothelin (ET-1) system is upregulated in obesity, leading to vasoconstriction and vascular damage. We hypothesized that in human obesity, a link might exist between changed circulating levels of vasoactive adipokines and ET-1-dependent vasoconstriction; **Methods**: We compared plasma concentrations of selected adipokines (Luminex assay) and the vasoactive response to blockade of endothelin type A receptors (ET_A_) by BQ-123 (strain-gauge plethysmography) in lean and obese individuals; **Results**: Plasma levels of adipokines with deleterious vascular actions, such as chemerin, visfatin, adipsin, and leptin, were higher in obese than in lean subjects (all *p* < 0.05). In contrast, circulating adiponectin, an adipokine with vasoprotective properties, showed no difference between groups (*p* > 0.05). The blood flow response to BQ-123 was greater in obese subjects than in lean subjects (*p* < 0.001), indicating an obesity-associated enhancement in ET-1-mediated vasoconstriction. In the entire population, circulating chemerin showed a direct correlation with the vasodilator response to BQ-123 (r = 0.30; *p* = 0.01). In contrast, no significant correlation was observed between concentrations of other adipokines and the response to BQ-123 (all *p* > 0.05). **Conclusions**: In human obesity, a direct link exists between increased circulating chemerin and augmented ET-1-mediated vasoconstriction. This observation contributes to explaining the detrimental vascular actions of chemerin and supports the view that targeting this adipokine might help prevent obesity-related vasculopathy.

## 1. Introduction

The cellular and structural remodeling of adipose tissue (AT) occurring in obesity to accommodate the excess fat burden is associated with inflammatory cell infiltration and oxidative stress [1]. These changes lead to adipose tissue dysfunction characterized by altered secretion of adipokines, with a specific upregulation of pro-inflammatory molecules [1]. This obesity-related dysregulation in adipokine expression, in turn, may affect several biological functions regulated by adipose tissue, including appetite, immunity, inflammation, and glucose and lipid metabolism [2]. Interestingly, as adipokine receptors are widely expressed on the surface of vascular endothelial and/or smooth muscle cells [3], changes in the circulating levels of these molecules might also impact vascular homeostasis [4,5,6].

Among various adipokines, chemerin, a chemoattractant protein known to recruit immune cells to sites of tissue damage [7], is also involved in other biological functions relevant to human disease, such as adipogenesis, glucose homeostasis, and energy balance [8]. Interestingly, chemerin has been shown to induce endothelial dysfunction by impairing the nitric oxide (NO) pathway [9], thereby contributing to the development of atherosclerosis, diabetes, and pre-eclampsia [10]. Similarly, visfatin, a multifaceted adipokine upregulated in obesity and diabetes, promotes endothelial dysfunction through inflammatory mechanisms [11] and plays a role in the destabilization of atherosclerotic plaques [3]. Moreover, adipsin, a protein of the complement system (complement factor D) secreted by adipocytes at higher rates in obesity [12,13], has been shown in epidemiological studies to bear a cross-sectional relationship with inflammation, endothelial dysfunction, and cardiovascular disease [14]. In addition, elevated circulating leptin in obesity is associated with high blood pressure, thrombosis, and a higher risk of cardiovascular disease [15]. In contrast, low levels of adiponectin are linked with increased prevalence of obesity-associated cardiovascular disorders and detrimental cardiovascular outcomes; therefore, strategies aimed at increasing the adiponectin/leptin ratio have been proposed as a therapeutic approach to cardiovascular dysfunction [16].

It is well established that vascular dysfunction at the early stages is characterized by changes in the main vasoactive mediators within the arterial wall, including the endothelin-1 (ET-1) system [17]. Studies performed by us and others in the human vasculature in vivo have consistently demonstrated that the ET-1 system is activated in overweight and obese adults, especially in those with metabolic abnormalities, independently of other cardiovascular risk factors [18,19]. This condition of endothelial dysfunction is crucial in the pathogenesis of atherosclerosis [20] and is an independent predictor of cardiovascular events [21,22].

We hypothesize that changes in plasma levels of vasoactive adipokines in human obesity might relate to increased ET-1-mediated vasoconstriction. To this end, we compared circulating levels of chemerin, visfatin, adipsin, leptin, and adiponectin and ET-1-mediated vasoconstrictor activity in lean subjects and obese individuals.

## 2. Materials and Methods

The data underlying the results of the present study can be made available by the corresponding author upon reasonable request.

### 2.1. Recruited Subjects

This study included lean subjects (body mass index (BMI) < 25 kg/m^2^) and individuals with central obesity (waist circumference ≥ 102 cm for males or ≥88 cm for females). Individuals with past or present evidence of cardiovascular disease (coronary artery disease, cerebrovascular or peripheral occlusive arterial disease, coagulopathy, vasculitis) or any other systemic condition were excluded from the study. In obese subjects on therapy with antihypertensive and/or lipid-lowering drugs, treatment was discontinued for at least one week before the study. Throughout this period, blood pressure was checked multiple times and, if required, treatment was resumed, leading to the exclusion of the patient from the study. All participants were non-smokers, and they were instructed to avoid alcohol and caffeinated beverages for at least 24 h prior to the study. Additionally, none of the participants were using vitamin supplements or participating in regular exercise programs. The study protocols received approval from the Institutional Review Board of Catholic University (approval n. 100356/16), and all participants provided written informed consent before their inclusion in the study.

### 2.2. Laboratory Analyses

Participants were instructed to fast for a minimum of 8 h prior to the study. Subsequently, while the participant lay supine, a 20-gauge catheter (Abbott Laboratories, Abbott Park, IL, USA) was inserted in a deep antecubital vein for blood collection. Blood samples were drawn in citrated tubes, rapidly spun in a refrigerated centrifuge, and then quickly stored at −80 °C. The aliquots used for this study were thawed immediately before the assay and thoroughly examined to confirm the absence of significant hemolysis or preservation issues. Measurements of insulin, chemerin, visfatin, adipsin, leptin, and adiponectin were carried out on the preserved plasma samples using Luminex assays (R&D Systems, Minneapolis, MN, USA).

### 2.3. Measurement of ET-1-Dependent Vascular Tone

The study involved a drug infusion into the brachial artery, with assessment of changes in forearm blood flow by strain-gauge venous occlusion plethysmography. The hospital’s research pharmacy prepared the investigational drug utilized in this investigation, adhering to specific protocols to guarantee the solutions’ bioavailability and sterility. Participants were placed in a supine position, and a 20-gauge Teflon catheter (Arrow Inc., Limerick, PA, USA) was inserted into the brachial artery of the non-dominant arm (typically the left) for drug infusion. A mercury-filled strain gauge fitted around the widest part of the forearm was then connected to a plethysmograph (model EC-6, Hokanson Inc., Bellevue, WA, USA), calibrated to assess the percent change in volume. A cuff was placed on the upper arm and inflated to 40 mm Hg using a rapid cuff inflator (model E-10, Hokanson) to restrict venous outflow from the limb for each measurement. Before each measurement, a wrist cuff was inflated to supra-systolic pressures for one minute to eliminate hand circulation. Flow measurements were taken for about 7 s every 15 s, and a total of 7 readings were taken for each average value. A standard mercury manometer was used for blood pressure measurement. The evaluation of ET-1-dependent vasoconstrictor tone was performed using BQ-123 (Bachem, Bubendorf, Switzerland), a selective blocker of type A endothelin (ET_A_) receptors, administered at a rate of 10 nmol/min (10 nmol/mL solution), which has been shown to block the vasoconstrictor effect of endogenous ET-1 in humans [23]. Selective blockade of ET_A_, rather than combined blockade of both ET_A_ and ET_B_ endothelin receptors, was used because we have previously observed that ET-1-dependent vasoconstriction is almost entirely mediated by ET_A_ receptors in insulin-resistant humans [24]. BQ-123 was infused for 60 min, and forearm flow was recorded at baseline (before administration of BQ-123) and every 10 min during administration of BQ-123. Percent changes in forearm blood flow from baseline at 60 min of BQ-123 infusion were considered as the response to ET_A_ antagonism. Throughout the study, infused volumes were balanced by administering different quantities of saline.

### 2.4. Statistical Analyses

Group comparisons were conducted using an unpaired *t*-test, whereas within-patient comparisons were performed utilizing a paired *t*-test. Univariate and multivariate analyses of association across the combined groups (lean and obese participants) were evaluated using standard linear regression analysis. In cases where the data did not follow a normal distribution, non-parametric tests were used, or the data were transformed into ranks before analysis. All probability values reported are two-tailed, and a *p* value of less than 0.05 has been considered statistically significant. Group data are presented as mean ± SEM.

## 3. Results

The baseline anthropometric, hemodynamic, and biochemical profiles of the participants are reported in Table 1, below. All obese participants had a waist circumference greater than the ATPIII criteria for central obesity [25], thereby indicating the presence of visceral adiposity.

### 3.1. Group Differences in Circulating Adipokines

**Figure 1:**
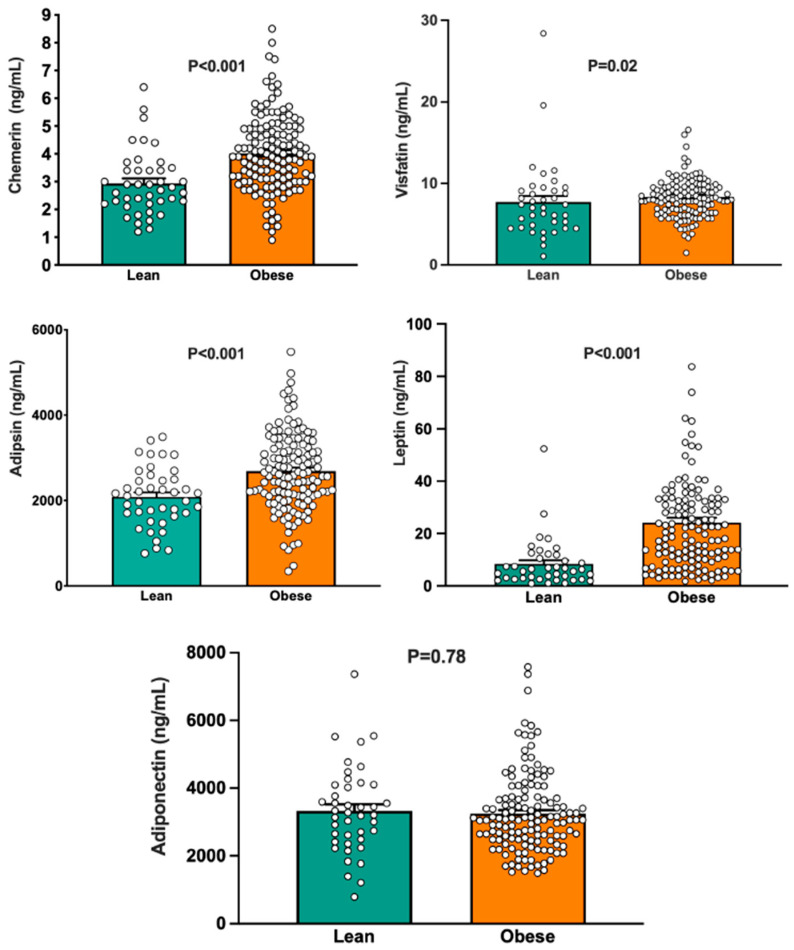
Graphs depicting circulating concentrations of chemerin (**top left panel**), visfatin (**top right panel**), adipsin (**middle left panel**), leptin (**middle right panel**), and adiponectin (**bottom panel**) in lean and obese subjects. The *p* values refer to the comparisons between the two groups by unpaired Student’s *t*-test. Bar values are means ± SEM.

The levels of chemerin in circulation were found to be greater in obese individuals compared to lean individuals (Figure 1, top left panel). Likewise, plasma visfatin levels were elevated in obese participants compared to lean ones (Figure 1, top right panel). Additionally, the levels of adipsin in circulation were higher in obese subjects relative to lean individuals (Figure 1, middle left panel). As expected, plasma leptin levels were significantly increased in obese individuals compared to their lean counterparts (Figure 1, middle right panel). In contrast, the circulating levels of adiponectin did not show a significant difference between the two groups (Figure 1, bottom panel).

### 3.2. Group Differences in Endothelin-Mediated Vasoconstrictor Tone

**Figure 2:**
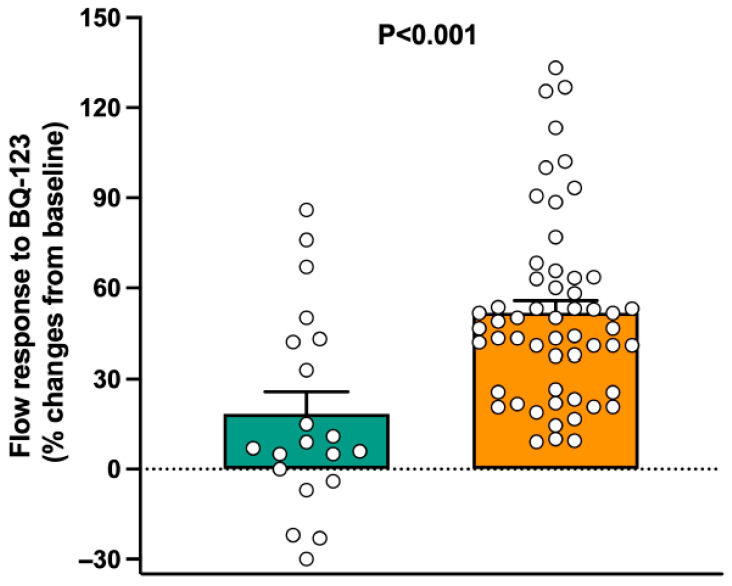
Graph showing the forearm blood flow responses to the ET_A_ receptor antagonist BQ-123 infused for 60 min in lean and obese subjects. The *p* values refer to the comparison between the 2 groups by unpaired Student’s *t*-test. Bar values are means ± SEM.

Forearm vascular reactivity to selective ET_A_ antagonism was measured in 20 lean subjects and 55 obese subjects. In lean subjects, at the end of the 60 min infusion of BQ-123, forearm blood flow was not significantly different from baseline (from 4.1 ± 0.4 to 4.8 ± 0.5 mL/min/dL; *p* = 0.06). In contrast, a significant vasodilator response from baseline was observed in obese individuals following administration of BQ-123 (from 3.8 ± 0.2 to 5.8 ± 0.3 mL/min/dL; *p* < 0.001). As a result, the increase in forearm blood flow from baseline following blockade of the ET_A_ receptor was greater in obese participants than in lean ones (Figure 2).

### 3.3. Relationships of the Vascular Response to BQ-123 with Circulating Adipokines and with Anthropometric, Hemodynamic, and Biochemical Variables

**Figure 3:**
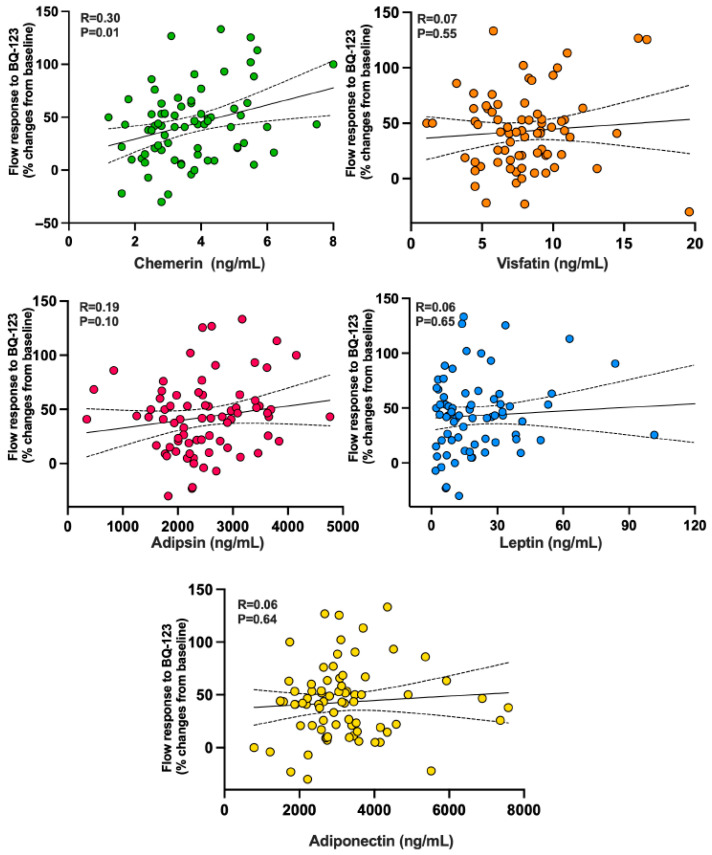
The graphs illustrate the linear associations found across the entire population between forearm blood flow responses to BQ-123 and levels of circulating chemerin (**top left panel**), visfatin (**top right panel**), adipsin (**middle left panel**), leptin (**middle right panel**), and adiponectin (**bottom panel**). The R values represent the regression coefficients, while the *p* values show the significance levels from the linear regression analysis.

In the whole population, the vasodilator response to ET_A_ receptor blockade had a linear, direct association with circulating chemerin (Figure 3, top left panel) but not with visfatin (Figure 3, top right panel) or adepsin (Figure 3, middle left panel). Also, no significant correlation was found between the vasodilator response to BQ-123 and plasma levels of leptin (Figure 3, bottom right panel) or adiponectin (Figure 3, bottom panel).

The vasodilator response to blockade of ET_A_ receptors exhibited a linear, direct relationship with BMI (R = 0.25; *p* = 0.04) but not with mean arterial pressure (R = 0.17; *p* = 0.17), plasma insulin (R = 0.07; *p* = 0.54), plasma glucose (R = 0.08; *p* = 0.53), HDL-cholesterol (R = 0.01; *p* = 0.96), or triglycerides (R = 0.21; *p* = 0.08).

In a backward stepwise regression analysis that considered chemerin, BMI, mean arterial pressure, plasma insulin, plasma glucose, HDL-cholesterol, and triglycerides as independent variables and the forearm flow response to BQ-123 as the dependent variable, chemerin emerged as the sole variable that showed an independent association with the vasodilator response to ET_A_ receptor blockade (R = 0.32; *p* = 0.009).

### 3.4. Relationships of Chemerin with Other Adipokines and with Demographic, Hemodynamic, and Biochemical Variables

**Figure 4:**
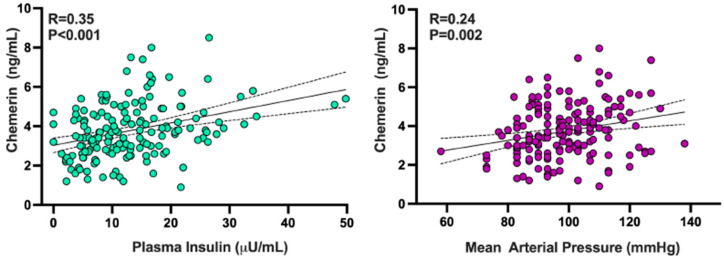
Graphs illustrating the linear associations between circulating chemerin and plasma insulin (**left panel**) and blood pressure (**right panel**) in the total population. The R values represent the regression coefficients, while the *p* values denote the significance level in the linear regression analysis.

Plasma chemerin concentrations had a linear direct relationship with those of adipsin (R = 0.19; *p* = 0.02) and leptin (R = 0.23; *p* = 0.002) but not with those of visfatin (R = 0.02; *p* = 0.84) and adiponectin (R = 0.10; *p* = 0.18). Circulating chemerin showed a linear direct association with plasma insulin (Figure 4, left panel), mean arterial pressure (Figure 4, right panel), BMI (R = 0.22; *p* = 0.005), and triglycerides (R = 0.21; *p* = 0.007) but not with plasma glucose (R = 0.10; *p* = 0.21). An inverse linear association was observed between plasma chemerin and HDL-cholesterol (R = 0.19; *p* = 0.02).

In a backward stepwise regression analysis that considered BMI, mean arterial pressure, plasma insulin, plasma glucose, HDL-cholesterol, and triglycerides as independent variables while using chemerin as the dependent variable, insulin showed the highest linear relationship with circulating chemerin (R = 0.38; *p* < 0.001), with mean arterial pressure preserving a weaker, independent association (*p* = 0.02).

## 4. Discussion

In our study, the circulating levels of various adipokines with known adverse vascular effects, such as chemerin, visfatin, adipsin, and leptin, were elevated in obese individuals compared to their lean counterparts. These findings are in keeping with those of previous studies, consistently reporting an upregulation of these adipokines in human obesity [1,3]. In contrast, circulating levels of adiponectin, an adipokine known to exert protective vascular actions, were not different between lean and obese individuals. Previous studies measuring circulating adiponectin levels in obese humans have yielded conflicting results, as some investigators have observed lower plasma adiponectin in obese subjects than in controls [26], while others have reported unchanged [27] or even increased [28] adiponectin levels in obesity. The precise reasons for these discrepancies are unclear, even though a likely explanation is the fact that the rate of adiponectin release from adipocytes depends on the “quality”, rather than the quantity, of adipose tissue [16]. In particular, the location of adipose tissue depots seems to exert a key influence on circulating adiponectin levels, as higher secretion rates of this peptide are observed in subcutaneous fat pads compared to visceral fat pads [29,30]; therefore, a heterogeneity of the clinical characteristics of the participants in different studies might explain their discrepant findings. Other possible explanations, however, include differences between studies in participant demographics known to influence circulating adiponectin (age, race, sex), smoking habits, and changes in the clearance rate of the peptide [16].

The novel finding of our study is the positive relationship observed between circulating chemerin concentrations and the vasodilator response to ET_A_ receptor blockade. At odds with the chemerin results, none of the other adipokines measured in our study were found to be linearly associated with the vasodilator response to ET_A_ antagonism. This observation suggests that mechanisms other than activation of the ET-1 system are likely involved in the adverse effects of visfatin, adipsin, and leptin in the human vasculature.

Increased vasodilator responsiveness to BQ-123 was observed in our obese participants compared to lean subjects, in keeping with previous data indicating that human obesity is characterized not only by defective endothelium-dependent vasodilator function but also by increased ET-1-mediated vasoconstrictor tone [18,19]. Taken in conjunction with the observed increase in circulating chemerin, this finding suggests that either common mechanisms underlie the combined increases in chemerin and ET-1 activity or chemerin plays a mechanistic role in the augmented ET-1-mediated vasoconstriction in human obesity. The latter possibility is supported by the results of our regression analyses, showing that chemerin remained the only independent variable linearly associated with the vasodilator effect of ET_A_ antagonism in the multiple regression model. Among all variables showing a linear association with circulating chemerin in our study population, plasma insulin had the strongest positive relationship. This observation confirms the findings of a recent study, reporting that plasma chemerin concentrations are elevated in patients with type 2 diabetes and those with insulin resistance compared to insulin-sensitive individuals [31]. Unfortunately, the cross-sectional design of our study does not allow deterministic insights into the cause-and-effect relationship between chemerin and hyperinsulinemia. However, previous data demonstrating that chemerin impairs insulin signaling by acting on several proteins of its cascade [32] suggest that upregulated chemerin is the determinant of hyperinsulinemia and insulin resistance. Regarding the potential mechanisms underlying the increased plasma chemerin concentrations found in our obese participants, their higher BMI and central distribution of fat were the most likely determinants of this phenomenon [33].

Several experimental studies have demonstrated that chemerin incites different vasoconstrictor mechanisms in the arterial wall. Lobato et al. reported that chemerin augments the vascular reactivity to ET-1 via activation of the MEK-ERK1/2 pathway in rat aortic rings [34]. Uncoupling of eNOS, with increased generation of reactive oxygen species and impaired NO/cGMP signaling, was subsequently reported by the same group of investigators as an additional mechanism of the endothelial dysfunction elicited by chemerin in the rat aorta [9] and in human microvascular endothelial and smooth muscle cells via its chemokine-like receptor 1 (CMKLR1 or ChemR23) [35]. In addition, Hanthazi et al. demonstrated that chemerin potentiates ET-1-induced vasoconstriction in isolated rat pulmonary arteries [36]. The independent association found in our study between plasma chemerin levels and blood pressure values extends the experimental evidence regarding the vasoconstrictor actions of chemerin, hinting that upregulation of vascular chemerin signaling plays a role in obesity-related hypertension. Support for this view comes from studies by Kennedy et al., demonstrating that chemerin contracts human saphenous veins and resistance arteries ex vivo and increases blood pressure when infused in rats [37], and by Ferland et al., showing that chemerin induces contraction in the rat aorta by activation of L-type Ca^2+^ channels [38]. Again, the design of our study does not allow us to obtain cause-and-effect insights in this regard. Also, the inherent limitations of a study performed in the intact human circulation in vivo do not allow us to ascertain the molecular mechanisms of the increased ET-1-dependent vasoconstriction in the context of elevated plasma chemerin, as observed in obese individuals.

Despite these limitations, however, our results support the notion that circulating chemerin may act as a biomarker for risk stratification in human obesity, as suggested by a previous demonstration that it independently predicts incident cardiovascular events. Thus, a prospective study, using data from a large cohort to evaluate the association between circulating chemerin and the risk of myocardial infarction, stroke, and type 2 diabetes, revealed a strong positive association between chemerin and cardiovascular events, independent of established risk factors, hence suggesting a potential role of chemerin in the development of cardiovascular disease [39].

## 5. Conclusions

Our study indicates that plasma concentrations of chemerin are elevated in obese patients and are positively related to ET-1 vasoconstrictor activity. As circulating chemerin may act as a biomarker of cardiovascular risk, selective targeting of this adipokine signaling might represent a promising strategy for cardiovascular prevention in obesity.

## Figures and Tables

**Table 1:** Clinical characteristics of the study population.

	Lean Subjects (*n* = 42)	Obese Patients (*n* = 134)	*p* Value
Sex, m/f	19/23	80/64	-
Age, yr	42 ± 2	41 ± 1	>0.05
BMI, kg/m^2^	23 ± 1	38 ± 1	<0.001
Waist, cm		118 ± 2	
MAP, mmHg	86 ± 1	95 ± 1	<0.001
Glucose, mg/dL	86 ± 1	101 ± 1	<0.001
Total Cholesterol, mg/dL	173 ± 4	196 ± 4	0.005
LDL Cholesterol, mg/dL	109 ± 5	125 ± 4	0.002
HDL Cholesterol, mg/dL	51 ± 2	45 ± 1	0.004
Triglycerides, mg/dL	92 ± 8	152 ± 8	<0.001
Insulin, μU/mL	7 ± 1	14 ± 1	<0.001
HOMA	1.5 ± 0.1	3.2 ± 0.3	<0.001

Data are expressed as mean ± SEM. Comparisons were performed by unpaired *t*-test. BMI, body mass index; MAP, mean arterial pressure; LDL, low-density lipoprotein; HDL, high-density lipoprotein; HOMA, HOMA index of insulin resistance.

## Data Availability

The data presented in this study are available on request from the corresponding author due to privacy.

## References

[1] Taylor E.B. (2021). The complex role of adipokines in obesity, inflammation, and autoimmunity. Clin. Sci..

[2] Unamuno X., Gomez-Ambrosi J., Rodriguez A., Becerril S., Fruhbeck G., Catalan V. (2018). Adipokine dysregulation and adipose tissue inflammation in human obesity. Eur. J. Clin. Investig..

[3] Akoumianakis I., Antoniades C. (2017). The interplay between adipose tissue and the cardiovascular system: Is fat always bad?. Cardiovasc. Res..

[4] Guzik T.J., Skiba D.S., Touyz R.M., Harrison D.G. (2017). The role of infiltrating immune cells in dysfunctional adipose tissue. Cardiovasc. Res..

[5] Koenen M., Hill M.A., Cohen P., Sowers J.R. (2021). Obesity, adipose tissue and vascular dysfunction. Circ. Res..

[6] Galley J.C., Singh S., Awata W.M.C., Alves J.V., Bruder-Nascimento T. (2022). Adipokines: Deciphering the cardiovascular signature of adipose tissue. Biochem. Pharmacol..

[7] Yan Y., Wang L., Zhong N., Wen D., Liu L. (2024). Multifaced roles of adipokines in endothelial cell function. Front. Endocrinol..

[8] Helfer G., Wu Q.F. (2018). Chemerin: A multifaceted adipokine involved in metabolic disorders. J. Endocrinol..

[9] Neves K.B., Lobato N.S., Lopes R.A., Filgueira F.P., Zanotto C.Z., Oliveira A.M., Tostes R.C. (2014). Chemerin reduces vascular nitric oxide/cGMP signalling in rat aorta: A link to vascular dysfunction in obesity?. Clin. Sci..

[10] Tan L., Lu X., Danser A.H.J., Verdonk K. (2023). The role of chemerin in metabolic and cardiovascular disease: A literature review of its physiology and pathology from a nutritional perspective. Nutrients.

[11] Romacho T., Valencia I., Ramos-Gonzalez M., Vallejo S., Lopez-Esteban M., Lorenzo O., Cannata P., Romero A., San Hipolito-Luengo A., Gomez-Cerezo J.F. (2020). Visfatin/eNampt induces endothelial dysfunction in vivo: A role for Toll-Like Receptor 4 and NLRP3 inflammasome. Sci. Rep..

[12] Dare A., Chen S.Y. (2024). Adipsin in the pathogenesis of cardiovascular diseases. Vascul. Pharmacol..

[13] Milek M., Moulla Y., Kern M., Stroh C., Dietrich A., Schon M.R., Gartner D., Lohmann T., Dressler M., Kovacs P. (2022). Adipsin serum concentrations and adipose tissue expression in people with obesity and type 2 diabetes. Int. J. Mol. Sci..

[14] Jin S., Eussen S., Schalkwijk C.G., Stehouwer C.D.A., van Greevenbroek M.M.J. (2023). Plasma factor D is cross-sectionally associated with low-grade inflammation, endothelial dysfunction and cardiovascular disease: The Maastricht study. Atherosclerosis.

[15] Kang K.W., Ok M., Lee S.K. (2020). Leptin as a key between obesity and cardiovascular disease. J. Obes. Metab. Syndr..

[16] Zhao S., Kusminski C.M., Scherer P.E. (2021). Adiponectin, leptin and cardiovascular disorders. Circ. Res..

[17] Bohm F., Pernow J. (2007). The importance of endothelin-1 for vascular dysfunction in cardiovascular disease. Cardiovasc. Res..

[18] Schinzari F., Iantorno M., Campia U., Mores N., Rovella V., Tesauro M., Di Daniele N., Cardillo C. (2015). Vasodilator responses and endothelin-dependent vasoconstriction in metabolically healthy obesity and the metabolic syndrome. Am. J. Physiol. Endocrinol. Metab..

[19] Weil B.R., Westby C.M., Van Guilder G.P., Greiner J.J., Stauffer B.L., DeSouza C.A. (2011). Enhanced endothelin-1 system activity with overweight and obesity. Am. J. Physiol. Heart Circ. Physiol..

[20] Sutton G., Pugh D., Dhaun N. (2019). Developments in the role of endothelin-1 in atherosclerosis: A potential therapeutic target?. Am. J. Hypertens..

[21] Jankowich M., Choudhary G. (2020). Endothelin-1 levels and cardiovascular events. Trends Cardiovasc. Med..

[22] Yang C., Zhu C.G., Guo Y.L., Wu N.Q., Dong Q., Xu R.X., Wu Y.J., Qian J., Li J.J. (2024). Prognostic value of plasma endothelin-1 in predicting worse outcomes in patients with prediabetes and diabetes and stable coronary artery diseases. Diabetes Metab. J..

[23] Tesauro M., Schinzari F., Rovella V., Di Daniele N., Lauro D., Mores N., Veneziani A., Cardillo C. (2009). Ghrelin restores the endothelin 1/nitric oxide balance in patients with obesity-related metabolic syndrome. Hypertension.

[24] Cardillo C., Campia U., Bryant M.B., Panza J.A. (2002). Increased activity of endogenous endothelin in patients with type II diabetes mellitus. Circulation.

[25] Grundy S.M., Cleeman J.I., Daniels S.R., Donato K.A., Eckel R.H., Franklin B.A., Gordon D.J., Krauss R.M., Savage P.J., Smith S.C. (2005). Diagnosis and management of the metabolic syndrome: An American Heart Association/National Heart, Lung, and Blood Institute Scientific Statement. Circulation.

[26] Silha J.V., Krsek M., Skrha J.V., Sucharda P., Nyomba B.L., Murphy L.J. (2003). Plasma resistin, adiponectin and leptin levels in lean and obese subjects: Correlations with insulin resistance. Eur. J. Endocrinol..

[27] Hoffstedt J., Arvidsson E., Sjolin E., Wahlen K., Arner P. (2004). Adipose tissue adiponectin production and adiponectin serum concentration in human obesity and insulin resistance. J. Clin. Endocrinol. Metab..

[28] Doumatey A.P., Bentley A.R., Zhou J., Huang H., Adeyemo A., Rotimi C.N. (2012). Paradoxical hyperadiponectinemia is associated with the metabolically healthy obese (MHO) phenotype in African Americans. J. Endocrinol. Metab..

[29] Turer A.T., Khera A., Ayers C.R., Turer C.B., Grundy S.M., Vega G.L., Scherer P.E. (2011). Adipose tissue mass and location affect circulating adiponectin levels. Diabetologia.

[30] Guenther M., James R., Marks J., Zhao S., Szabo A., Kidambi S. (2014). Adiposity distribution influences circulating adiponectin levels. Transl. Res..

[31] Zhao L., Zhou J., Abbasi F., Fathzadeh M., Knowles J.W., Leung L.L.K., Morser J. (2024). Chemerin in participants with or without insulin resistance and diabetes. Biomedicines.

[32] Leniz A., Gonzalez M., Besne I., Carr-Ugarte H., Gomez-Garcia I., Portillo M.P. (2022). Role of chemerin in the control of glucose homeostasis. Mol. Cell. Endocrinol..

[33] Rourke J.L., Dranse H.J., Sinal C.J. (2013). Towards an integrative approach to understanding the role of chemerin in human health and disease. Obes. Rev..

[34] Lobato N.S., Neves K.B., Filgueira F.P., Fortes Z.B., Carvalho M.H., Webb R.C., Oliveira A.M., Tostes R.C. (2012). The adipokine chemerin augments vascular reactivity to contractile stimuli via activation of the MEK-ERK1/2 pathway. Life Sci..

[35] Neves K.B., Nguyen Dinh Cat A., Lopes R.A., Rios F.J., Anagnostopoulou A., Lobato N.S., de Oliveira A.M., Tostes R.C., Montezano A.C., Touyz R.M. (2015). Chemerin regulates crosstalk between adipocytes and vascular cells through Nox. Hypertension.

[36] Hanthazi A., Jespers P., Vegh G., Degroot G.N., Springael J.Y., Lybaert P., Dewachter L., Mc Entee K. (2019). Chemerin influences endothelin- and serotonin-induced pulmonary artery vasoconstriction in rats. Life Sci..

[37] Kennedy A.J., Yang P., Read C., Kuc R.E., Yang L., Taylor E.J., Taylor C.W., Maguire J.J., Davenport A.P. (2016). Chemerin elicits potent constrictor actions via chemokine-like receptor 1 (CMKLR1), not G-protein-coupled receptor 1 (GPR1), in human and rat vasculature. J. Am. Heart Assoc..

[38] Ferland D.J., Darios E.S., Neubig R.R., Sjogren B., Truong N., Torres R., Dexheimer T.S., Thompson J.M., Watts S.W. (2017). Chemerin-induced arterial contraction is G(i)- and calcium-dependent. Vascul. Pharmacol..

[39] Eichelmann F., Schulze M.B., Wittenbecher C., Menzel J., Weikert C., di Giuseppe R., Biemann R., Isermann B., Fritsche A., Boeing H. (2019). Chemerin as a biomarker linking inflammation and cardiovascular diseases. J. Am. Coll. Cardiol..

